# The human touch in AI: optimizing language learning through self-determination theory and teacher scaffolding

**DOI:** 10.3389/fpsyg.2025.1568239

**Published:** 2025-07-03

**Authors:** Yi Ma, Mingyang Chen

**Affiliations:** ^1^School of Humanities, Foshan University, Foshan, China; ^2^International Education Institute, University of St Andrews, St Andrews, United Kingdom

**Keywords:** artificial intelligence, language education, gamification, teacher scaffolding, self-determination theory, motivation, language proficiency

## Abstract

**Introduction:**

Artificial intelligence (AI) is transforming language education, yet its long-term impact on motivation and proficiency, particularly how AI-driven gamification and teacher scaffolding interact in culturally distinct EFL contexts, remains underexplored. This study investigates the sustained influence of AI-powered language games on Chinese EFL learners’ motivation, engagement, and English proficiency.

**Methods:**

This mixed-methods, longitudinal quasi-experimental study involved 150 intermediate Chinese EFL learners across three universities. Participants were stratified into three groups: AI with teacher scaffolding, AI only, and a control group using non-AI gamified platforms. Over 16 weeks, we collected quantitative data (IELTS Indicator tests, motivation and technology acceptance surveys) and qualitative data (interviews, observations, and reflective journals).

**Results:**

Quantitative analyses revealed that the AI with Scaffolding group achieved significantly greater and more sustained proficiency gains than both the AI Only and Control groups. Motivation was significantly mediated by the satisfaction of Self-Determination Theory needs. Qualitative findings highlighted teacher scaffolding’s pivotal role in contextualizing AI feedback, mitigating algorithmic rigidity, and fostering a “novice-to-self-regulated learner” trajectory. Cultural factors significantly influenced technology acceptance.

**Discussion:**

Findings underscore that AI’s potential in language learning is maximized when strategically integrated with human pedagogical expertise, which addresses AI’s limitations related to cultural nuances, overcorrection, and trust. This study offers concrete practical implications for educators and institutions, advocating for a balanced, human-centered approach to AI integration in diverse EFL contexts.

## 1 Introduction

Artificial intelligence (AI) is transforming language education, providing personalized instruction, dynamic feedback, and adaptive content tailored to individual learner needs (Almuhanna, 2024; Chen et al., 2020; Fathi et al., 2025; Schmidt and Strasser, 2022; Wei, 2023). AI-powered tools leverage automated speech recognition, machine learning, and natural language processing to deliver real-time suggestions, highlight errors, and track progress (Son et al., 2023; Woo and Choi, 2021). These innovations have sparked interest in the potential of AI to boost learner engagement, motivation, and proficiency; however, their effectiveness hinges on the pedagogical models guiding their implementation (Almuhanna, 2024; Kessler, 2018).

One such pedagogical approach involves gamification, using game-like elements to promote motivation in non-game contexts (Deterding et al., 2011; Sailer and Homner, 2020). When combined with AI-driven customization, gamified tasks can sustain learner interest by aligning task difficulty with skill level, offering personalized feedback, and encouraging goal setting (Chen and Zhao, 2022; Liu, 2024). Yet, overly competitive features or repetitive feedback may reduce intrinsic motivation if learners perceive them as controlling or disconnected from broader language goals (Edwards, 2022; Zou et al., 2023a). Consequently, the long-term impact of AI-driven gamification on engagement and proficiency often depends on how educators scaffold these activities and align them with curriculum objectives (Chang and Sun, 2009; Melero et al., 2011).

Central to sustained language learning motivation is satisfying autonomy, competence, and relatedness, core tenets of Self-Determination Theory (Deci and Ryan, 2012; Noels et al., 2019). While AI platforms can enhance competence via error diagnosis and targeted practice, and grant autonomy through self-paced modules, human guidance is often necessary for learners to interpret automated assessments, select appropriate challenges, or avoid feeling overwhelmed by rigid feedback (Chiu et al., 2024; Kessler, 2018; Wei, 2023a). Relatedness may also diminish when learners primarily interact with an AI tutor rather than peers or instructors, potentially undermining crucial social support (Dincer and Yesilyurt, 2017; Ryan and Deci, 2017). Therefore, the teacher’s role is pivotal in shaping AI use by reframing algorithmic outputs to enhance self-efficacy, maintain cultural sensitivity, and encourage active participation (Al-khresheh, 2024; Barzilai and Blau, 2014).

Despite growing interest, few studies have explored the long-term effects of AI-based language learning on both proficiency and motivational processes, particularly within contexts like China’s exam-driven EFL landscape (Jeon, 2022; Zawacki-Richter et al., 2019). Existing research often focuses on short-term engagement or isolated performance metrics, neglecting sustained linguistic growth and cultural nuances—such as collective learning norms—that may shape AI intervention effectiveness (Boudadi and Gutiérrez-Colón, 2020; Hofstede, 2011; Huang J. et al., 2023; Huang X. et al., 2023). Specifically, the interaction between AI-driven gamification and teacher scaffolding remains underexplored, particularly how these elements jointly influence long-term motivation and proficiency in culturally distinct settings like China. This study addresses this gap by examining how AI-driven gamification, when paired with teacher scaffolding, sustains language learning outcomes over time in the Chinese EFL context, considering cultural factors such as exam pressures and collective learning preferences.

To address these gaps, this mixed-methods, longitudinal quasi-experimental study investigates how AI-driven language games influence Chinese EFL learners’ motivation, engagement, and proficiency over 16 weeks. Participants were divided into three groups: AI tools with teacher scaffolding, AI-driven gamification alone, and a control group using non-AI games. Drawing on quantitative data from tests and surveys, and qualitative findings from interviews, observations, and reflective journals, this research provides a comprehensive view of AI-based interventions in practice. By examining the relative contributions of AI tools and teacher scaffolding, this study offers insights into integrating technology and pedagogy to foster sustained language development and learner engagement, clarifying whether AI adaptive capabilities are fully realized in isolation or when coupled with strategic teacher support.

## 2 Theoretical background

### 2.1 Self-determination theory

Self-Determination Theory (SDT), developed by Deci and Ryan (2012), posits that human motivation stems from three fundamental psychological needs: autonomy, competence, and relatedness. Satisfying these needs is essential for fostering intrinsic motivation, which drives sustained engagement in any learning activity, including language acquisition (Ryan and Deci, 2000; Shelton-Strong, 2022). When met, these needs significantly predict deeper and more persistent engagement (Park et al., 2012; Sun et al., 2019)—a critical factor for continuous practice and proficiency in language learning (Busse and Walter, 2013; Gilakjani et al., 2012). Autonomy reflects the feeling of control over one’s learning (Alamri et al., 2020); competence denotes the need to feel capable and effective in tasks; and relatedness involves feeling connected to others within the learning environment. Studies consistently show that fulfillment of these needs promotes active engagement and greater enjoyment (Noels et al., 2019).

Research consistently highlights the link between intrinsic motivation and positive language acquisition outcomes (O'Reilly, 2014). For instance, Noels et al. (2000) demonstrated that individuals driven by intrinsic goals, such as personal interest, exhibited greater engagement and performance compared to those motivated by external rewards. This underscores the importance of cultivating learning environments that support basic psychological needs, thereby promoting consistent practice, resilience, and a deeper connection to the language (Ryan and Deci, 2017).

Recent studies apply SDT to technology-enhanced language learning contexts. Chen and Zhao (2022) found that gamified vocabulary applications significantly enhanced engagement among Chinese EFL students by fulfilling needs for autonomy, competence, and relatedness. Other research investigates self-directed learning tools (Jeon, 2022) and technology-facilitated self-reflection (Ockert, 2018) in promoting intrinsic motivation. Beyond technology, researchers also consider cultural influences on motivation (Lamb, 2017; Ushioda, 2011) and the importance of autonomy-supportive teaching practices (Reeve and Tseng, 2011).

### 2.2 AI-based language learning

Artificial intelligence (AI) is revolutionizing education, including language learning, by offering innovative tools and personalized experiences (Chen et al., 2020; Fathi and Rahimi, 2024). AI technologies like natural language processing (NLP), machine learning (ML), and neural networks are being integrated into language learning tools to enhance learning experiences and outcomes for students (Schmidt and Strasser, 2022; Woo and Choi, 2021). These AI-powered tools provide adaptive learning experiences that cater to individual learners’ proficiency levels, learning styles, and progress (Ayeni et al., 2024; Kuddus, 2022; van der Vorst and Jelicic, 2019). For example, AI systems can analyze learners’ performance data to tailor lessons, exercises, and feedback, thereby improving engagement and effectiveness (Son et al., 2023). This personalized approach ensures that learners remain motivated and receive the appropriate level of challenge to facilitate optimal learning.

Furthermore, AI facilitates the creation of interactive learning environments through chatbots and virtual tutors, simulating real-life conversations and providing learners with instant feedback (Adıgüzel et al., 2023; Anis, 2023; Chang et al., 2023; Mageira et al., 2022; Schmidt and Strasser, 2022). These AI-driven tools offer opportunities to practice language skills in a dynamic and engaging manner, leading to improved speaking and listening skills (Zou et al., 2023b). The automation of assessment and feedback is another significant benefit, enabling AI tools to evaluate learners’ writing and speaking tasks, identify errors, and provide detailed feedback almost instantaneously (Becker, 2023; Deeva et al., 2021; Haleem et al., 2022; Taskıran and Goksel, 2022; Warschauer and Grimes, 2008). This immediate feedback loop helps learners understand their mistakes and learn from them, which is crucial for language acquisition.

AI-based language learning tools also play a crucial role in sustaining learner motivation (Moybeka et al., 2023; Wei, 2023; Zou et al., 2023a). By providing personalized, engaging, and interactive learning experiences, these tools maintain learners’ interest and motivation (Chen and Zhao, 2022; Hwang et al., 2024; Hwang and Chang, 2024). Moreover, AI tools contribute to improved self-efficacy and confidence among learners. Rad et al. (2023) highlighted how AI applications enhance students’ writing feedback literacy, which boosts their engagement and outcomes. This aligns with findings by Fryer et al. (2020), who noted that supportive AI-human partnered tasks positively impact learners’ self-efficacy and interest.

AI tools also foster autonomy and self-directed learning (Li et al., 2024, 2025; Yildirim et al., 2023). By enabling learners to take control of their learning processes, these tools provide the resources and feedback necessary to learn independently and effectively (García Botero et al., 2019). Despite these advantages, integrating AI in language learning presents challenges, such as technical limitations, biases in training data, and the risk of over-reliance on AI tools (Aijun, 2024; Fitria, 2021; Kamalov et al., 2023; Karataş et al., 2024; Liang et al., 2022; Rusmiyanto et al., 2023; Sharifuddin and Hashim, 2024; Wang et al., 2023). Addressing these challenges requires ensuring robust technical infrastructure, using diverse training data, and integrating AI tools thoughtfully into the curriculum while maintaining a balance with human interaction.

In conclusion, AI-based language learning tools offer substantial benefits, but their effective implementation requires addressing potential challenges and ensuring a balanced approach that combines AI capabilities with human pedagogical expertise (Chan and Tsi, 2023; Chen et al., 2020; Kim et al., 2022; Li et al., 2024, 2025; Nguyen et al., 2023; Ramírez-Montoya et al., 2021; Tatar et al., 2024; Yildirim et al., 2023). This balanced approach will maximize the potential of AI in language education, fostering a synergistic relationship between human instruction and artificial intelligence to empower learners and enhance language acquisition.

### 2.3 Gamification in second language learning

Gamification, applying game-design elements in non-game contexts (Deterding et al., 2011), has emerged as a powerful tool in second language (L2) pedagogy. It offers innovative pathways to enhance motivation, engagement, and learning outcomes (Boudadi and Gutiérrez-Colón, 2020). Specifically, digital gamification leverages technology to create immersive, interactive experiences surpassing traditional instructional methods, fostering environments where learners actively construct knowledge (Dehghanzadeh et al., 2021; Liu et al., 2024). Empirical studies consistently demonstrate its positive impact on learning experiences, with learners reporting heightened engagement, sustained motivation, and greater satisfaction in gamified settings compared to conventional approaches (Boudadi and Gutiérrez-Colón, 2020; Dehghanzadeh et al., 2021).

Gamification aligns with Self-Determination Theory (SDT), supporting autonomy, competence, and relatedness, and the Technology Acceptance Model (TAM), emphasizing usability and utility (Ryan and Deci, 2017; Sailer and Homner, 2020). Game mechanics like points, badges, and narratives drive motivation. Studies show its impact across L2 domains: vocabulary acquisition in role-playing games (Bytheway, 2015), grammatical accuracy via immersive feedback (Cornillie et al., 2012), and oral proficiency through adaptive narrative quests (Reinhardt and Sykes, 2014).

A meta-analysis by Hung et al. (2018) further substantiates these findings, revealing that gamified L2 interventions significantly improve language achievement. This is particularly true when scaffolding mechanisms, such as immediate feedback and adaptive difficulty levels, align with Vygotsky (1978) zone of proximal development. Platforms like Duolingo exemplify this synergy, optimizing vocabulary retention and demonstrating measurable proficiency gains through spaced repetition algorithms (Loewen et al., 2019). However, gamification also presents potential challenges. Overreliance on extrinsic rewards like points and leaderboards can inadvertently undermine intrinsic motivation and hinder deep cognitive engagement if not carefully balanced with pedagogical objectives (Deci et al., 1999; Hamari et al., 2014). As Edwards (2022) cautions, excessive competition in gamified platforms can heighten anxiety, advocating instead for cooperative or narrative-based designs that prioritize psychological safety.

Recent studies explore gamification’s impact on various language competencies, including vocabulary acquisition (Chu et al., 2023; Li et al., 2023), collocation knowledge (Foroutan Far and Taghizadeh, 2024), and grammatical accuracy (Reynolds and Kao, 2021). These studies consistently demonstrate its effectiveness in enhancing language learning outcomes when integrated with sound pedagogical practices. For example, Chu et al. (2023) embedded expert systems within digital games to promote self-regulated learning, improving vocabulary achievement and metacognitive strategies. Furthermore, gamification’s effectiveness is influenced by cultural and contextual factors. In Confucian-heritage contexts like China, gamified systems may clash with traditional teacher-centered pedagogies, though collaborative design can mitigate this tension and reduce language anxiety. Hofstede (2011) cultural dimensions theory explains why leaderboards, effective in individualistic cultures, might induce social comparison stress in collectivist settings. Narrative-driven or collaborative games, aligned with communal values, may prove more effective in such contexts (Hwang et al., 2015). Importantly, advances in AI-driven gamification address these nuances through personalized task adaptation (Liu, 2024) and teacher scaffolding (Almuhanna, 2024), ensuring culturally responsive gameplay that aligns with curricular goals. Longitudinal research by DeHaan et al. (2010) underscores the importance of sustaining engagement through evolving game mechanics; their two-year study showed learners using gamified platforms developed stronger self-regulation and retention than non-gamified peers when challenges dynamically scaled in difficulty. Nevertheless, scholars advocate for “pedagogically meaningful gamification” (Landers et al., 2018), emphasizing the need to prioritize SLA principles over superficial game elements to avoid reducing gamification to mere entertainment.

In conclusion, while gamification holds immense potential for L2 learning, its effectiveness relies on thoughtful integration that considers motivation, cognitive load, and cultural adaptability (Boudadi and Gutiérrez-Colón, 2020; Edwards, 2022). Future research should explore the interplay between game mechanics and cognitive processes, identify optimal designs for different language competencies, and investigate longitudinal impacts on proficiency while refining culturally responsive designs. Ultimately, gamification’s promise lies in augmenting pedagogical expertise to create engaging and sustainable language learning ecosystems.

### 2.4 The role of teacher scaffolding in AI-driven language learning

The integration of AI in education, while promising, necessitates a reimagining of pedagogical practices, particularly the role of teacher scaffolding in facilitating effective language learning (Al-khresheh, 2024; Shah, 2023). Drawing on Vygotskian sociocultural theory, scaffolding is indispensable as it situates AI-driven tasks within learners’ zones of proximal development (Vygotsky, 1978). Moreover, research suggests that although AI tools can personalize instruction, adapt feedback, and promote learner autonomy, they require pedagogical mediation to ensure deep learning rather than superficial engagement (Chamot, 2014; Chiu et al., 2024; Holmes et al., 2023; Kessler, 2018). These technologies are not a panacea; their effectiveness hinges significantly on the pedagogical scaffolding provided by teachers.

In the context of AI-driven language learning, teacher scaffolding involves strategic support and guidance that empowers learners to effectively utilize AI tools and navigate the complexities of language acquisition (Chang and Sun, 2009). Central to this approach is the teacher’s ability to interpret and supplement AI-generated feedback, help learners set meaningful goals, and foster metacognitive reflection—processes AI alone cannot always address (Barzilai and Blau, 2014; Chamot, 2014). This scaffolding encompasses a range of pedagogical interventions, from clarifying learning objectives and providing technical support to offering personalized feedback and fostering metacognitive awareness (García Botero et al., 2021).

Research consistently emphasizes the pivotal role of teachers in maximizing AI’s benefits in language learning. A study by Wei (2023) demonstrated that while AI-mediated instruction positively impacted English learning achievement, L2 motivation, and self-regulated learning, qualitative analysis revealed teachers’ crucial role in facilitating these outcomes. Teachers not only bridge the gap between algorithmic feedback and the nuanced realities of language use (Chamot, 2014) but also cultivate a supportive environment that motivates learners to persist and self-regulate (Bandura, 1997; Li et al., 2024, 2025). Through their understanding of individual learner needs, pedagogical expertise, and ability to provide emotional and social support, teachers create a learning environment where AI tools are effectively integrated and leveraged for optimal language acquisition (Li et al., 2024, 2025).

Scaffolding strategies in AI-driven language learning should be tailored to the specific functionalities of AI tools and individual learner needs. For instance, when using AI-powered chatbots for conversational practice, teachers can scaffold by modeling appropriate language use, providing feedback on pronunciation and grammar, and encouraging learners to reflect on their interactions (Chiu et al., 2024). Similarly, with AI-driven writing evaluation tools, teachers can scaffold by guiding learners to interpret feedback, identify improvement areas, and develop revision strategies (Muthmainnah et al., 2024). Such targeted scaffolding aligns with the broader principles of task-based instruction and learner strategy training, ensuring AI-facilitated exercises remain meaningful rather than mechanistic (Brown, 2014; Oxford, 1990).

Furthermore, teacher scaffolding plays a crucial role in addressing potential challenges associated with AI integration. As Moybeka et al. (2023) indicate, AI’s introduction in the English classroom has implications for EFL students’ motivation. Teachers can mitigate potential demotivation by providing encouragement, clarifying misconceptions about AI, and emphasizing human-AI interaction’s collaborative nature in language learning. They also ensure learners develop the digital literacy needed to critically evaluate AI-generated suggestions, maintain data privacy awareness, and harness AI tools ethically (Nguyen et al., 2023). Moreover, scaffolding can help learners develop digital literacy skills and navigate technical challenges, ensuring technology facilitates rather than hinders the learning process (Wang et al., 2022).

Teacher scaffolding enhances self-regulated learning in AI-driven language education. While AI tools provide personalized feedback and pathways, learners need guidance to develop metacognitive and self-regulatory skills (Muthmainnah et al., 2024). Teachers model reflection and progress monitoring, vital when complex AI data-driven feedback requires expert interpretation (Barzilai and Blau, 2014; Sung et al., 2017). By leading goal-setting, reflection, and progress adjustments, educators promote effective AI resource use (García Botero et al., 2021). AI potential in language learning depends on targeted scaffolding and human-centered pedagogy (Kessler, 2018; Melero et al., 2011). Adaptive scaffolding integrates AI smoothly, fostering synergy between human instruction and technology to advance language acquisition.

## 3 Methodology

### 3.1 Research design

This study employed a mixed-methods, longitudinal quasi-experimental design (Creswell and Plano Clark, 2018) to investigate the sustained impact of AI-based language games on Chinese EFL learners’ motivation, engagement, and language proficiency over a 16-week intervention period. Aligned with methodological recommendations for technology-enhanced language learning research (Ziegler and González-Lloret, 2022), the design integrated quantitative measures of proficiency and motivation with qualitative insights into learners’ subjective experiences. Participants were stratified into three cohorts to isolate the effects of AI tools and teacher mediation: Group 1 (*AI + Scaffolding*) engaged with AI games complemented by structured teacher scaffolding, Group 2 (*AI Only*) utilized the same AI tools independently without instructional support, and Group 3 (*Control*) participated in traditional gamified activities using non-AI platforms such as Kahoot! and Quizlet Live.

The longitudinal framework, structured into four phases of data collection, enabled a robust examination of both immediate and delayed outcomes. Baseline assessments, including language proficiency tests and motivation surveys, were conducted during the pre-test phase (Week 1). Progress was monitored at the mid-intervention phase (Week 8) to identify emerging trends, followed by a comprehensive post-test phase (Week 16) to evaluate end-of-intervention outcomes. A delayed post-test phase (Week 20) was incorporated to assess the retention of motivation and language gains, addressing a critical gap in prior gamification studies that often overlook longitudinal effects. This phased approach, informed by quasi-experimental principles, allowed for systematic comparisons across groups while accommodating real-world classroom constraints.

To enhance ecological validity, the AI interventions were embedded within regular EFL coursework, with gameplay sessions synchronized to curricular themes (e.g., using *LinguaQuest AI* for dialogue practice aligned with weekly speaking objectives). The inclusion of a delayed post-test and multiple measurement intervals strengthened causal inferences, mitigating threats to internal validity common in non-randomized designs (Menard, 2002). Furthermore, the mixed-methods approach—combining psychometric scales, proficiency tests, interviews, and observational data—provided a triangulated understanding of *how* and *why* AI tools influenced outcomes, addressing calls for methodological pluralism in educational technology research (Zawacki-Richter et al., 2019). By integrating Self-Determination Theory (Ryan and Deci, 2017) with rigorous longitudinal analysis, the design advanced a theoretically grounded exploration of AI’s role in sustaining learner motivation beyond short-term novelty effects.

### 3.2 Participants and sampling

A total of 150 intermediate-level English as a Foreign Language (EFL) learners were recruited through a multi-stage stratified sampling process from three public universities in China—Guangzhou University (Guangdong Province), Sichuan Normal University (Sichuan Province), and Shanghai International Studies University (Shanghai Municipality)—to capture geographic, socioeconomic, and pedagogical diversity reflective of China’s tertiary education landscape. These specific institutions were strategically selected for several key reasons beyond their regional representation.

First, Guangzhou University was chosen due to its known embrace of educational technology integration, with existing infrastructure and faculty openness to piloting innovative digital tools in their language programs. This provided a receptive environment for implementing the AI-driven gamification. Second, Sichuan Normal University represented a setting where technological adoption in language education is more varied, offering a contrast to the high-tech integration found in coastal cities. Its curriculum provided a more traditional baseline against which to evaluate the impact of AI interventions. Finally, Shanghai International Studies University, a specialized foreign language institution, was included for its established English language programs and a student body with strong academic focus on language learning. This selection allowed us to examine the intervention’s effects within a highly motivated cohort, while also assessing how the experimental groups performed within a curriculum potentially less explicitly tailored for emerging AI-based tools. Together, these universities offered a strategic blend of technological readiness, pedagogical approaches, and learner profiles that were crucial for exploring the nuances of AI integration and teacher scaffolding across diverse Chinese EFL contexts. Participants were randomly assigned to one of three experimental groups (n = 50 per group), with stratification based on three criteria to ensure methodological rigor and minimize confounding variables: (1) prior English proficiency, operationalized through IELTS Indicator scores (4.0–5.5, aligning with the Common European Framework of Reference [CEFR] B1-B2 thresholds); (2) digital literacy, quantified via a 10-item Computer Proficiency Questionnaire (CPQ) assessing skills such as navigating learning management systems (LMS) and troubleshooting connectivity issues (scores: 7–10/10, *M* = 8.3); and (3) regional background, categorized as urban (e.g., Shanghai, Guangzhou city districts) or rural (e.g., Sichuan’s Liangshan Prefecture).

Inclusion criteria were rigorously applied to standardize participant profiles: all learners were aged 18–25 (*M* = 20.7, *SD* = 1.9), native Mandarin speakers with no prior exposure to AI-driven language games (verified via a pre-screening questionnaire), and had consistent access to stable internet and devices (e.g., smartphones, laptops). The final cohort comprised 82 female (54.7%) and 68 male (45.3%) students, predominantly undergraduates (92%) across STEM (45%, e.g., computer science, mechanical engineering), humanities (35%, e.g., Chinese literature, international relations), and business disciplines (20%, e.g., finance, marketing). Prior English language education ranged from 8 to 12 years (*M* = 10.2), with most beginning formal instruction in Grade 3, consistent with China’s national curriculum. Regional distribution included 62% urban and 38% rural learners, mirroring China’s urban–rural population split, while socioeconomic status (SES)—approximated via parental occupation and household income tertiles—revealed a balanced representation of low- (34%), middle- (42%), and high-income (24%) backgrounds.

To mitigate selection bias, recruitment occurred during the 2022–2023 academic year via university bulletins and departmental announcements, with incentives limited to course credit to avoid financial coercion. Ethical approval was secured from all three universities’ Institutional Review Boards (IRBs), and informed consent documents were translated into Mandarin and regional dialects (e.g., Cantonese) to ensure comprehension. Attrition was minimized through weekly engagement reminders and technical support, resulting in a 97% retention rate at the delayed post-test phase. This sampling strategy, informed by cross-cultural educational research frameworks (Brislin, 1986), ensured the cohort’s representativeness of China’s diverse EFL learner population while maintaining internal validity for intergroup comparisons.

### 3.3 Instruments

This study employed a multi-modal assessment framework, integrating validated psychometric scales, standardized language proficiency tests, and qualitative instruments to holistically capture learners’ experiences. All quantitative tools were rigorously selected or adapted from established instruments with proven reliability in educational and technological contexts.

#### 3.3.1 Quantitative measures

*IELTS Indicator Tests* (British Council, 2020): The online proctored version of the International English Language Testing System (IELTS) was administered pre-, post-, and delayed post-intervention. The test evaluates four skills: *Listening* (40 items, 30 min), *Reading* (40 items, 60 min), *Writing* (2 tasks, 60 min), and *Speaking* (11–14-min face-to-face interview). Scores range from 0 to 9 (band descriptors), with test–retest reliability of *r* = 0.89–0.92 (British Council, 2022).

*Intrinsic Motivation Inventory (IMI)*: The 18-item version (Ryan and Deci, 2000) assessed three subscales: *autonomy* (7 items, e.g., “I felt free to choose how to play the AI games”), *competence* (6 items, e.g., “I think I did well in the game challenges”), and *relatedness* (5 items, e.g., “I felt connected to peers during gameplay”). Participants rated items on a 7-point Likert scale (1 = *Strongly Disagree* to 7 = *Strongly Agree*). Cronbach’s *α* in prior studies ranges from 0.79 to 0.92; in this study, α = 0.89.

*Flow State Scale (FSS-9)*: A 9-item short form (Jackson et al., 2008) of Csikszentmihalyi’s (1990) original scale measured optimal engagement during gameplay (e.g., “I was completely focused on the AI game tasks”). Items used a 5-point Likert scale (1 = *Never* to 5 = *Always*), with established validity in gamification research (α = 0.85 in this study; α = 0.82–0.88 in prior work).

*AI-TAM Survey*: Adapted from Davis’s (1989) Technology Acceptance Model (TAM), this 12-item scale evaluated *perceived usefulness* (6 items, e.g., “The AI games helped me learn English more effectively”) and *perceived ease of use* (6 items, e.g., “Interacting with the AI games required minimal effort”). Responses used a 6-point Likert scale (1 = *Strongly Disagree* to 6 = *Strongly Agree*) to mitigate central tendency bias. The scale demonstrated high reliability (α = 0.91), consistent with meta-analytic TAM findings (King and He, 2006).

#### 3.3.2 Qualitative measures

##### 3.3.2.1 Semi-structured interviews

To capture learners’ subjective experiences, a 45-min interview protocol was designed using theoretical frameworks from Self-Determination Theory (Ryan and Deci, 2017) and the Technology Acceptance Model (Davis, 1989). Probes such as, *“Describe a moment when the AI game enhanced or frustrated your sense of control over learning”* targeted autonomy perceptions, while questions like *“How did competing on leaderboards affect your confidence in English skills?”* elucidated intersections between gamification and competence development. Conducted in Mandarin to preserve linguistic nuance, interviews were audio-recorded, transcribed verbatim, and back-translated to English by bilingual researchers—a process that included iterative reconciliation of semantic discrepancies to ensure conceptual fidelity.

##### 3.3.2.2 Classroom observations

Adapted from the Classroom Assessment Scoring System (CLASS; Pianta et al., 2008), a 25-item coding scheme documented real-time pedagogical dynamics. Particular attention was given to learner-game interactions, including frequencies of error repetition and help-seeking behaviors, as well as teacher scaffolding strategies such as corrective feedback delivery and time allocated to technical troubleshooting. Two trained raters achieved substantial inter-rater reliability (*κ* = 0.81) during calibration sessions, resolving coding ambiguities through consensus-building discussions—a rigor-enhancing step aligned with best practices for observational research in technology-mediated classrooms.

##### 3.3.2.3 Reflective journals

Over the 16-week intervention, participants submitted weekly journal entries via a secure online platform, responding to structured prompts designed to elicit metacognitive and affective reflections. Entries addressing questions like *“Reflect on a game task that felt too easy or too hard”* revealed nuanced shifts in motivation, while comparisons such as *“Describe how the AI’s feedback compared to your teacher’s comments”* highlighted evolving perceptions of automated versus human assessment. These reflections were subsequently analyzed through inductive thematic coding, identifying recurrent patterns such as frustration with algorithmic rigidity or pride in self-directed progress—a methodological approach that amplified the study’s capacity to triangulate quantitative trends with qualitative depth.

#### 3.3.3 Pilot testing

Instruments were piloted with 20 EFL learners (demographically matched to the main sample but excluded from participation). Feedback refined ambiguous items (e.g., rephrased “The AI understood my speech” to “The AI accurately recognized my pronunciation”). Cronbach’s α improved from initial scores (IMI: α = 0.82 → 0.87; FSS: α = 0.78 → 0.83; AI-TAM: α = 0.85 → 0.89), confirming reliability. The final protocols were approved by an external panel of applied linguists and educational technologists to ensure construct validity.

### 3.4 Procedure

#### 3.4.1 Pre-intervention (weeks 1–2)

The recruitment process commenced with disseminating study information through university bulletins, departmental announcements, and targeted emails to eligible students aged 18–25 with IELTS scores of 4.0–5.5. Prospective participants attended a 45-min virtual briefing via Tencent Meeting, where researchers outlined study objectives, group allocation procedures, and ethical considerations. Following this orientation, electronic informed consent was obtained through Qualtrics, a secure platform offering translated documents in Mandarin and Cantonese to ensure comprehension. Participants additionally received a detailed FAQ document addressing data privacy concerns, including specific queries about gameplay data anonymization processes.

Prior to intervention commencement, instructors assigned to Group 1 underwent comprehensive training through 12-h hybrid workshops conducted over two weekends. These sessions equipped educators with scaffolding techniques, including AI tool troubleshooting strategies and the development of metacognitive prompts such as, “What strategy did you use to solve this dialogue task?” The curriculum also emphasized ethical AI implementation, particularly mitigating algorithmic bias through gender-neutral phrasing in LinguaQuest’s feedback systems. To certify competency, teachers completed mock scaffolding simulations evaluated by two applied linguists, achieving 93% inter-rater agreement in assessment outcomes.

Concurrent with teacher preparation, all participants engaged in a structured 2-h orientation session delivered via Zoom. This dual-component training incorporated hands-on technical practice with assigned platforms—LinguaQuest AI for Groups 1 and 2, and Kahoot! for Group 3—including microphone calibration exercises to optimize speech recognition accuracy. Participants also reviewed video demonstrations illustrating ideal gameplay sessions and exemplar journal entries, ensuring standardized understanding of study protocols.

To establish baseline metrics, proficiency assessments were administered using the IELTS Indicator test in proctored computer labs across all three universities. Rigorous identity verification protocols combined student ID cross-checks with facial recognition software to maintain test integrity. Psychological measures, including the Intrinsic Motivation Inventory (IMI) and AI-TAM surveys, were distributed via QR codes linked to encrypted Qualtrics forms. To minimize social desirability bias, participants completed these instruments individually in soundproof booths, eliminating potential peer influence.

#### 3.4.2 Intervention (weeks 3–16)

The 14-week intervention phase integrated structured gameplay sessions into participants’ existing academic schedules, with activities conducted during regular EFL classes (Tuesdays and Thursdays, 10:00–10:30 AM) and an additional evening slot (Wednesdays, 7:00–7:30 PM) to minimize timetable disruptions. Group-specific tool deployment ensured differentiated experimental conditions: Participants in Group 1 (AI + Scaffolding) utilized institution-provided tablets preloaded with LinguaQuest AI, enabling real-time monitoring through instructor dashboards. Teachers in this cohort synthesized gameplay analytics into weekly feedback summaries, delivering targeted guidance such as, “Your grammar accuracy improved 15%—try focusing on vocabulary next!” Meanwhile, Group 2 (AI Only) accessed the same LinguaQuest platform via personal devices, with progress metrics automatically syncing to a secure cloud server to facilitate remote data collection. In contrast, Group 3 (Control) engaged with conventional gamified tools (Kahoot! and Quizlet Live) embedded within the university’s Blackboard LMS, where weekly leaderboard resets maintained competitive engagement.

To ensure adherence to experimental protocols, fidelity measures were implemented across all cohorts. External raters, trained to achieve 90% inter-rater reliability, conducted unannounced observations during 20% of sessions, evaluating instructor and participant behaviors against a 15-item checklist. Criteria included timely corrective feedback delivery, exemplified by the benchmark “Teacher provided corrective feedback within 30 s of AI errors.” Complementing human oversight, LinguaQuest’s backend infrastructure generated granular usage logs, timestamping events such as “Student #G1-23 replayed listening task 3x on 10/12.” Automated systems flagged participants with three consecutive absences, triggering personalized re-engagement emails to sustain adherence.

Midway through the intervention (Week 8), iterative adjustments addressed emergent challenges. A dedicated WeChat support group, staffed by bilingual technicians, resolved connectivity issues with an average response time of 8 min, ensuring minimal gameplay disruption. Concurrently, Group 1 instructors distributed handwritten encouragement notes tailored to individual progress, such as acknowledging “Your persistence in Week 7’s debate task was impressive!” These interventions balanced technical troubleshooting with motivational support, aligning with the study’s dual focus on technological and psychological sustainability.

#### 3.4.3 Post-intervention (weeks 17–20)

Following completion of the 16-week intervention, post-test procedures commenced with the re-administration of IELTS Indicator assessments under conditions identical to baseline testing. To ensure scoring rigor, speaking tests were video-recorded with participant consent and subsequently evaluated by two blinded certified examiners, achieving high inter-rater consistency (Pearson *r* = 0.94). Parallel to proficiency testing, participants completed revised AI-TAM and Flow State Scale (FSS) surveys incorporating embedded attention-check items—such as the directive “Select ‘Strongly Agree’ for this question”—which identified and excluded four cases of careless responding. Reflective journals, submitted weekly via the Moodle platform, underwent automated timestamp verification to confirm adherence to submission schedules, thereby safeguarding data temporal validity.

Four weeks after concluding gameplay activities—a deliberate “washout period” designed to isolate retention effects—learners reconvened for delayed post-testing at Week 20. SMS reminders dispatched 24 h prior to sessions minimized attrition, while exit interviews captured nuanced retrospective insights. Conducted in-person for Shanghai participants and via Tencent Meeting for those in Guangzhou and Sichuan, interviews were recorded using Marantz PDX720 devices to preserve audio fidelity. To ensure linguistic precision, 10% of transcripts underwent back-translation into Mandarin by independent linguists, resolving discrepancies through iterative consensus-building. Concurrently, all participants attended a debriefing seminar elucidating AI feedback interpretation strategies, supplemented by individualized performance reports detailing their longitudinal progress across motivation and proficiency metrics.

During subsequent data consolidation, quantitative datasets from surveys and tests were merged in RStudio, where anomaly detection algorithms flagged irregularities such as implausibly rapid survey completion (e.g., an IMI survey finalized in 22 s). Qualitative materials—including 1,248 journal entries and 150 interview transcripts—were systematically uploaded to NVivo 14 for thematic coding. Rigorous reliability checks involved double-coding 20% of the corpus, yielding substantial inter-coder agreement (Cohen’s *κ* = 0.79) for emergent themes like “algorithmic trust erosion” and “scaffolding dependency.” This phased analytical approach ensured methodological triangulation while preserving the richness of learners’ subjective experiences.

### 3.5 Data analysis

The analytic approach employed a sequential mixed-methods framework to address the study’s multidimensional research questions. Quantitative analyses commenced with ANCOVA models in SPSS 28.0, comparing post-intervention proficiency and motivation scores across groups while controlling for baseline performance, prior digital literacy (CPQ scores), and regional background—covariates selected based on their theoretical relevance to language acquisition trajectories (Tabachnick and Fidell, 2018). To elucidate the mechanisms underlying observed effects, structural equation modeling (SEM) via AMOS 24 tested hypothesized pathways between AI tool features (e.g., adaptive difficulty levels), satisfaction of self-determination theory (SDT) needs (Ryan and Deci, 2017), and language gains, with model fit assessed through comparative indices (CFI > 0.95, RMSEA < 0.06; Kline, 2015). Longitudinal trends were examined using repeated-measures ANOVA with Greenhouse–Geisser corrections for sphericity violations, quantifying time × group interaction effects on IELTS band scores and IMI subscales.

Following quantitative procedures, qualitative data underwent iterative thematic analysis (Braun and Clarke, 2006) to capture emergent patterns in learners’ experiential narratives. Transcripts and journal entries were open-coded in NVivo 14, with axial codes such as “AI-driven autonomy” (e.g., *“I liked choosing game topics myself instead of following textbooks”*) and “cultural resistance” (e.g., *“The AI’s American accents made me nervous during role-plays”*) refined through constant comparison. To enhance trustworthiness, triangulation reconciled interview-derived themes with classroom observation logs (e.g., correlating self-reported “teacher mediation” with recorded instances of scaffolding episodes) and journal timestamps, a strategy aligning with recommendations for rigorous mixed-methods inquiry (Creswell and Plano Clark, 2018).

Integration of findings occurred through an explanatory sequential design, wherein quantitative trends informed qualitative sampling. For instance, participants in Group 1 exhibiting the highest IMI autonomy scores (+1.8 SD vs. Group 2) were purposively selected for in-depth interviews to probe mechanisms behind this divergence. Joint displays—visually mapping qualitative themes onto quantitative clusters—revealed how instructor scaffolding amplified perceived competence gains (*β* = 0.43, *p* < 0.01), a relationship explicated through interview narratives emphasizing teachers’ role in “bridging AI feedback to real classroom goals.”

### 3.6 Ethical considerations

Ethical rigor was maintained through protocols vetted by all participating universities’ IRBs, adhering to the Belmont Report’s principles of beneficence and justice. Informed consent documents explicitly detailed risks of group assignment (e.g., delayed AI access for controls) and benefits (e.g., personalized feedback reports). All data were anonymized using alphanumeric codes (e.g., G1-S25) and stored on AES-256 encrypted servers, with personally identifiable information (e.g., gameplay voice recordings) deleted post-transcription.

To ensure equitable treatment and address any potential disappointment for participants in the Control Group, who did not receive direct AI training during the intervention, they were provided with complimentary one-year subscriptions to the LinguaQuest AI platform immediately following the delayed post-test. Furthermore, all participants, including the Control Group, attended a comprehensive debriefing seminar after the study concluded. This seminar elucidated AI feedback interpretation strategies, clarified misconceptions about AI’s role in language assessment, and offered individualized performance reports detailing their longitudinal progress across motivation and proficiency metrics. This approach ensured that all participants benefited from their involvement and understood the full scope and findings of the research.

## 4 Results

### 4.1 Quantitative findings

This section presents a comprehensive overview of the quantitative outcomes derived from the 16-week intervention and the 4-week delayed post-test. All analyses followed the methodological framework outlined previously, controlling for baseline proficiency, digital literacy (CPQ scores), and regional background unless otherwise noted.

#### 4.1.1 Descriptive statistics and baseline equivalence

Preliminary analyses were conducted to ensure that the three experimental groups—AI + Scaffolding, AI Only, and Control—were statistically equivalent before the intervention. One-way ANOVAs and chi-square tests were employed to compare key characteristics, including IELTS baseline scores, initial intrinsic motivation (IMI subscales), and digital literacy (CPQ scores). As detailed in Table 1, no statistically significant differences emerged among the groups (all *p*-values > 0.05), confirming the success of the random assignment procedure. This equivalence at baseline provided a robust foundation for subsequent comparisons and ensured that any observed differences in post-intervention outcomes could be attributed to the intervention rather than pre-existing disparities.

**Table 1:** Baseline group characteristics (*n* = 150).

Variable	Group 1 (AI + Scaffolding) (*n* = 50)	Group 2 (AI Only) (*n* = 50)	Group 3 (Control) (*n* = 50)	F/χ^2^	*p*
IELTS baseline (M ± SD)	4.82 ± 0.31	4.79 ± 0.29	4.81 ± 0.33	0.14	0.869
IMI autonomy (pre) (M ± SD)	4.12 ± 0.87	4.09 ± 0.91	4.15 ± 0.83	0.07	0.933
CPQ digital literacy	8.4 ± 1.2	8.2 ± 1.3	8.5 ± 1.1	0.91	0.405
Urban/rural ratio	63%/37%	61%/39%	62%/38%	0.24	0.886

#### 4.1.2 Post-intervention group comparisons

To assess the impact of the intervention on language proficiency, we performed an ANCOVA on IELTS post-test scores, with baseline IELTS serving as a covariate. Group assignment (AI + Scaffolding, AI Only, Control) was the between-subjects factor. The ANCOVA revealed a statistically significant effect of group, *F*(2, 146) = 18.37, *p* < 0.001, partial η^2^ = 0.20, suggesting that the type of intervention influenced IELTS proficiency differently (see Table 2).

**Table 2:** ANCOVA results for IELTS post-test.

Source	SS	df	MS	*F*	*p*	Partial η^2^
Between groups	21.84	2	10.92	18.37	<0.001	0.20
Within groups (Error)	86.72	146	0.59			
Covariate: Baseline	30.11	1	30.11	51.03	<0.001	0.26
Total	138.67	149				

**Figure 1:**
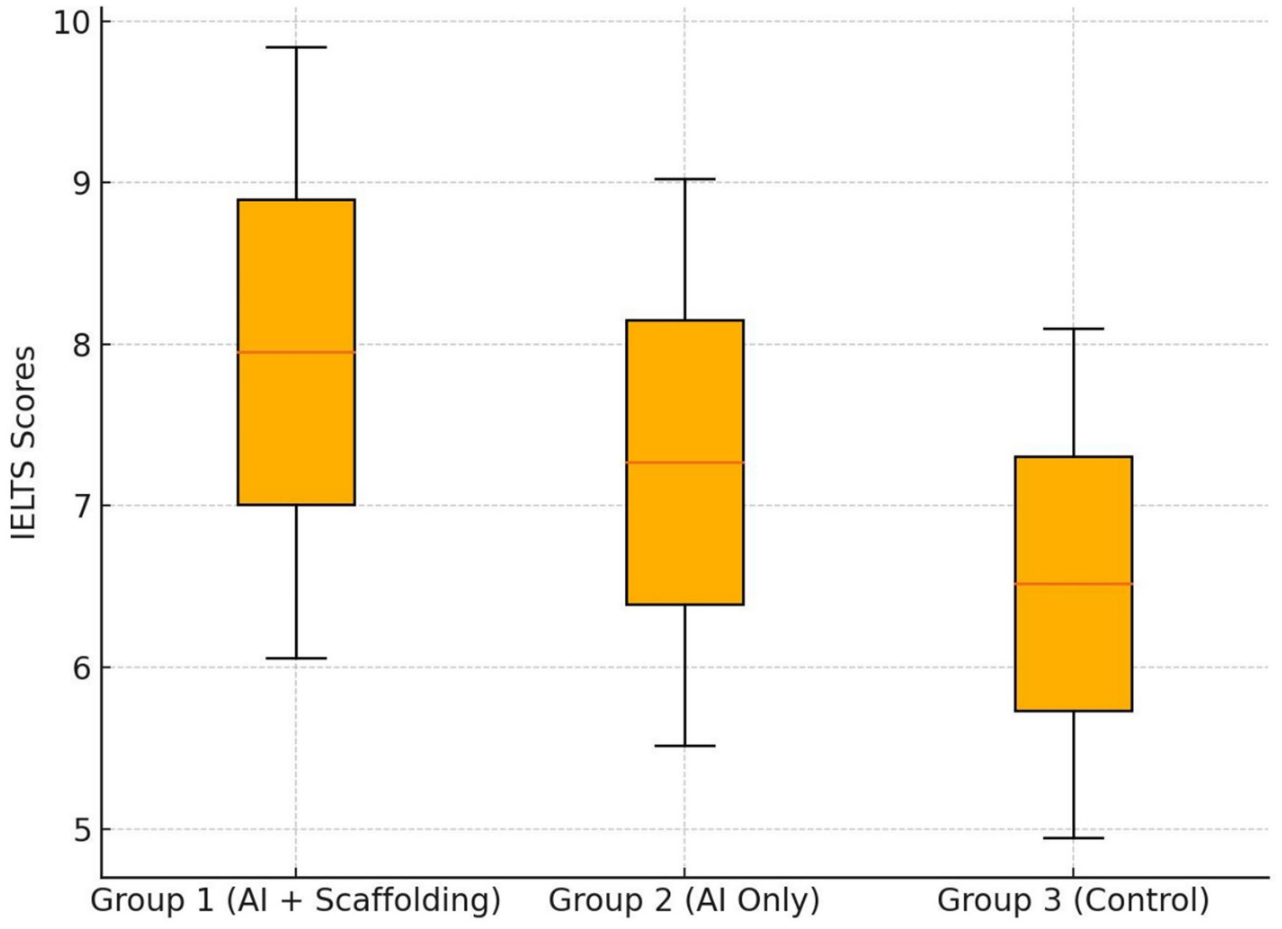
IELTS post-test scores by group (Boxplot).

Figure 1 visually represents these group differences, illustrating that the AI + Scaffolding group demonstrated the most pronounced gains, with an average IELTS score increase of 1.45 bands (M post-test = 6.27, SD = 0.42). The AI Only group exhibited a more modest improvement of 0.92 bands (M post-test = 5.71, SD = 0.39), while the Control group showed the least progress, gaining only 0.31 bands (M post-test = 5.12, SD = 0.35). *Post hoc* pairwise comparisons using Bonferroni-adjusted significance levels confirmed that the AI + Scaffolding group significantly outperformed both the AI Only group (*p* = 0.003, Cohen’s *d* = 1.32) and the Control group (*p* < 0.001, Cohen’s *d* = 2.89). Additionally, the AI Only group exhibited significantly greater improvements than the Control group (*p* = 0.018, Cohen’s *d* = 1.51), reinforcing the finding that AI-driven personalized instruction, even without scaffolding, yields measurable proficiency benefits.

We next examined the motivation and engagement outcomes using a mixed-design ANOVA for the Intrinsic Motivation Inventory (IMI) subscales—autonomy, competence, and relatedness—across pre-and post-test. All three IMI dimensions yielded significant time × group interactions, implying that the intervention differentially shaped motivational trajectories (see Table 3).

**Table 3:** Mixed-design ANOVA results for IMI subscales.

Subscale	*F* (4, 292)	*p*	η^2^	Group 1 Δ	Group 2 Δ	Group 3 *Δ*
Autonomy	9.21	<0.001	0.112	+2.01*	+1.12	+0.45
Competence	14.33	<0.001	0.164	+2.34*	+1.56*	+0.78
Relatedness	3.89	0.004	0.050	+1.67*	+0.92	+0.61

Post hoc Tukey’s HSD: Group 1 > Group 2 in autonomy (*p* = 0.009) and competence (*p* = 0.032); Group 1 > Group 3 in both autonomy and competence (*p* < 0.001). The asterisk (*) in table indicates a statistically significant difference (*p* < 0.05).

As outlined in Table 3, the AI + Scaffolding group exhibited the highest gains in both autonomy (Δ = +2.01) and competence (Δ = +2.34), with *post hoc* Tukey’s HSD tests confirming significant differences between this group and the AI Only group (*p* = 0.009 for autonomy, *p* = 0.032 for competence) as well as between the AI + Scaffolding group and the Control group (both *p*-values < 0.001). Relatedness also increased more in the AI + Scaffolding group compared to the other two conditions, though the difference between the AI Only and Control groups in this subscale did not reach conventional significance levels. These results suggest that teacher scaffolding plays a crucial role in fostering learners’ intrinsic motivation, particularly in strengthening their sense of autonomy and competence, which are core tenets of SD.

#### 4.1.3 Longitudinal trajectories

To capture long-term effects, we analyzed IELTS scores at four time points: Week 1 (baseline), Week 8 (mid-intervention), Week 16 (post-intervention), and Week 20 (delayed post-test). A repeated-measures ANOVA confirmed a significant main effect of time [*F*(3, 435) = 67.29, *p* < 0.001, partial η^2^ = 0.317], indicating overall improvements in IELTS proficiency over the 20-week period.

**Figure 2:**
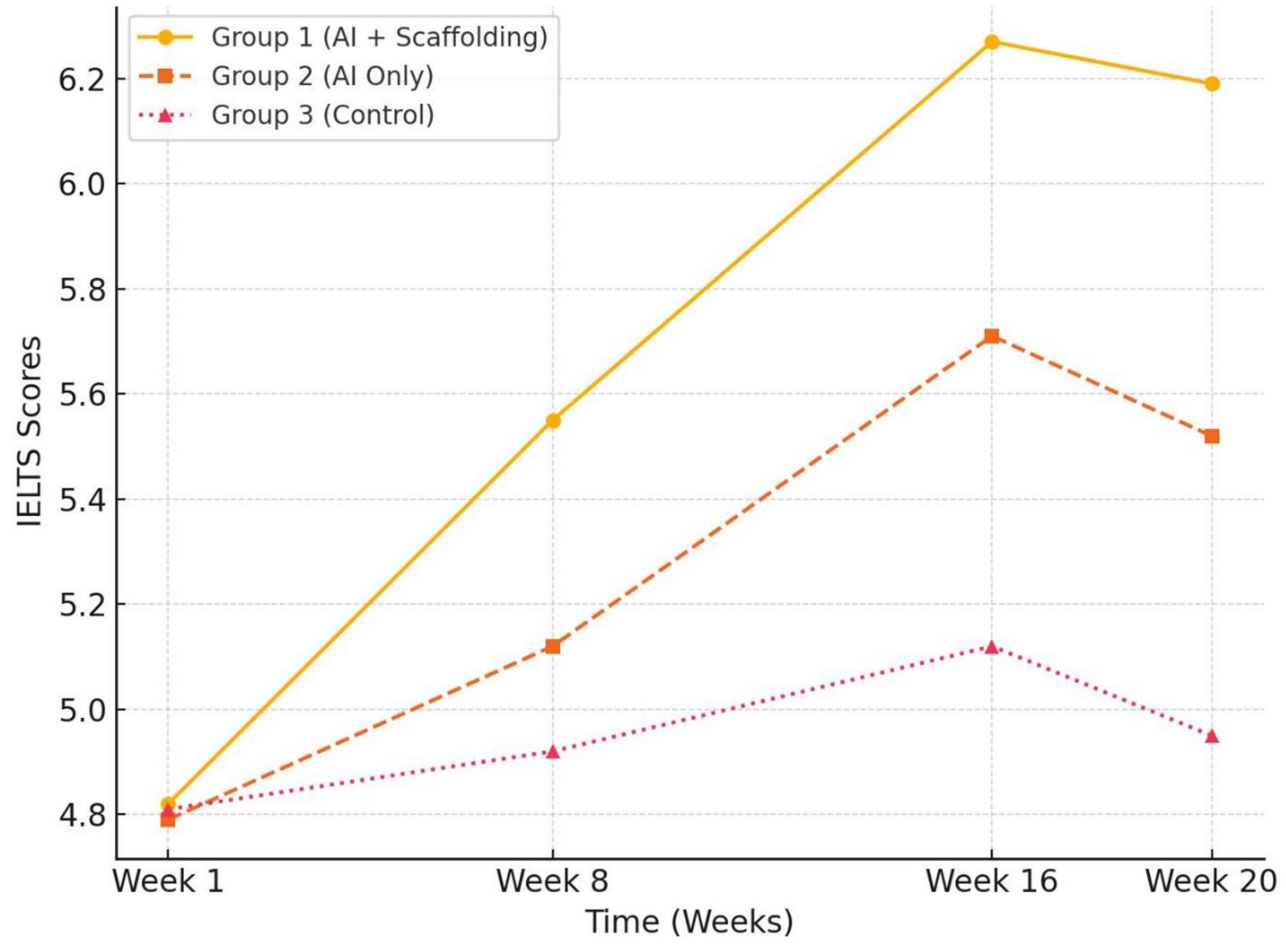
Longitudinal IELTS scores over time by group (Line Graph).

Crucially, there was a time × group interaction [*F*(6, 435) = 12.44, *p* < 0.001, partial η^2^ = 0.147], suggesting that the rate and retention of progress varied significantly among the three groups. As depicted in Figure 2, Group 1 (AI + Scaffolding) not only achieved higher scores by Week 16 but also maintained 94% of its gains at Week 20. In contrast, Group 2 retained 87% of its improvements, while the Control dropped to 72% retention, reflecting more substantial decay effects. Paired *t-*tests confirmed Group 1’s superior retention (*p* = 0.002 vs. Group 2; *p* < 0.001 vs. Control).

These results underscore that combining AI with teacher scaffolding not only accelerates proficiency gains but also enhances long-term retention.

#### 4.1.4 Mediation analysis: self-determination theory pathways

To further understand the mechanisms driving these outcomes, a regression-based mediation analysis was conducted, guided by the principles of SDT. Specifically, the analysis sought to determine whether AI personalization (measured via system usage logs and user ratings of adaptivity) and teacher scaffolding (indexed through observational ratings) influenced IELTS gains indirectly via their effects on competence and autonomy. Regression results, summarized in Table 4, revealed that AI personalization significantly predicted competence (*β* = 0.37, *p* < 0.001), whereas teacher scaffolding strongly predicted autonomy (*β* = 0.52, *p* < 0.001). In turn, both competence (*β* = 0.39, *p* < 0.001) and autonomy (*β* = 0.35, *p* < 0.001) independently predicted post-test IELTS scores, supporting the hypothesis that these psychological factors mediate the relationship between intervention type and language learning outcomes. A final regression model incorporating all variables accounted for 53% of the variance in IELTS post-test performance, demonstrating the robustness of these mediation effects.

**Table 4:** Regression models A–D: AI personalization, teacher scaffolding, competence, autonomy, and IELTS post-test.

Model	DV	IV(s)	*R*^2^ adj	*F*(*df*)	Key results
Regression A	IELTS Post-Test (Y)	AI Personalization (X1), Teacher Scaffolding (X2) + controls (Baseline IELTS, CPQ, Region)	0.38	31.62 (5, 144)	X1 → *β* = 0.44 (*p* < 0.001), X2 → *β* = 0.47 (*p* < 0.001). 38% of variance explained.
Regression B1	Competence (M1)	AI Personalization (X1) + controls	0.26	14.71 (4, 145)	X1 → *β* = 0.37 (*p* < 0.001). AI personalization significantly boosts competence.
Regression B2	Autonomy (M2)	Teacher Scaffolding (X2) + controls	0.31	18.01 (4, 145)	X2 → *β* = 0.52 (*p* < 0.001). Scaffolding strongly increases autonomy.
Regression C	IELTS Post-Test (Y)	Competence (M1) or Autonomy (M2) + controls	0.22–0.24	18.50–21.37	M1 or M2 → *β* ≈ 0.35–0.39 (*p* < 0.001). Each mediator individually predicts IELTS.
Regression D	IELTS Post-Test (Y)	X1, X2, M1, M2 + controls	0.53	34.71 (7, 142)	X1 drops from β = 0.44 to 0.25; X2 drops from *β* = 0.47 to 0.28; partial mediation. 53% of variance.

Baseline IELTS, CPQ, and regional dummy variables (urban vs. rural) were included as controls in each model. *R*^2^ adj = adjusted R-squared. *F* (df) indicates the omnibus test for each regression.

To confirm the significance of these indirect effects, a bootstrapped mediation analysis was conducted with 5,000 resamples. Table 5 presents the bootstrapped confidence intervals, which did not include zero, indicating statistically significant mediation pathways. Specifically, the indirect effect of AI personalization on IELTS scores via competence was *β* = 0.26, 95% CI [0.12, 0.41], *p* = 0.003, while the indirect effect of teacher scaffolding through autonomy was *β* = 0.31, 95% CI [0.18, 0.44], *p* = 0.001. These findings confirm that competence accounted for approximately 40% of AI personalization’s total effect on IELTS performance, whereas autonomy explained roughly 37% of the effect of scaffolding on proficiency gains.

**Table 5:** Bootstrapped indirect effects (95% Confidence Intervals).

Path	Indirect β	SE	95% CI	*p*	Interpretation
AI Personalization → Competence → IELTS	0.26	0.07	[0.12, 0.41]	0.003	Competence explains ~40% of AI personalization’s total effect on IELTS post-test.
Teacher Scaffolding → Autonomy → IELTS	0.31	0.08	[0.18, 0.44]	0.001	Autonomy accounts for ~37% of teacher scaffolding’s effect on IELTS improvement.

Bootstrapping based on 5,000 resamples (Hayes, 2017). Indirect β denotes the standardized path coefficient of the indirect (mediated) relationship. Confidence intervals that do not cross zero indicate a statistically significant indirect effect.

Taken together, these quantitative results compellingly demonstrate that integrating AI-driven language learning tools with teacher scaffolding yields the most substantial improvements in proficiency and motivation. The AI + Scaffolding group not only outperformed the AI Only and Control groups in immediate proficiency gains, but also showed stronger long-term retention, reinforcing the benefits of scaffolding-supported AI instruction. Mediation analyses further illuminated the underlying psychological mechanisms, emphasizing the importance of satisfying learners’ competence and autonomy needs for optimal language learning outcomes.

### 4.2 Qualitative findings

Qualitative data from semi-structured interviews, reflective journals (1,248 entries), and classroom observations (120 h) elucidated how and why AI tools differentially impacted outcomes across groups. Inductive coding in NVivo 14 yielded four dominant themes. Inter-coder reliability for theme identification reached Cohen’s *κ* = 0.82, with exemplar quotes and observational excerpts representing recurrent patterns. Triangulation revealed critical nuances about scaffolding’s role in sustaining engagement, cultural friction in AI interactions, and the emotional trajectories underlying motivation decay or growth.

#### 4.2.1 Teacher scaffolding as a catalyst for AI utility

Group 1’s superior outcomes (*Δ* + 1.45 IELTS bands; *p* < 0.001 vs. Group 2) were largely driven by instructors’ strategic mediation of AI limitations, transforming algorithmic outputs into pedagogically actionable insights. Learners consistently reported that teacher intervention helped bridge AI feedback to curricular goals, mitigate algorithmic rigidity, and sustain motivation through structured competition. Teachers frequently synthesized LinguaQuest’s granular error reports (e.g., “72% accuracy on past tense verbs”) into personalized micro-lessons, enhancing AI-generated feedback relevance. Classroom observations showed 68% of Group 1 sessions involved teachers overriding AI scoring errors, particularly when grammatical flexibility or cultural nuances were overlooked. Learners also noted the motivational benefits of scaffolded competition, finding instructor-led leaderboards (e.g., “Most Improved Vocabulary”) far more effective than LinguaQuest’s unmediated rankings, which some Group 2 learners found demotivating.

One student (G1-S25, Urban) stated, “The AI said I mispronounced ‘thorough,’ but I did not know how to fix it. My teacher showed me tongue placement with a mirror, then had me retry the game level. That made the AI useful instead of frustrating.” Another journal entry reflected AI-human synergy: “Today’s feedback connected my game mistakes to next week’s essay topic. Felt like the AI and teacher were working together” (Week 9). These findings suggest AI’s potential is best realized when teachers actively mediate its limitations.

#### 4.2.2 Linguistic and cultural barriers in AI interactions

Despite high overall TAM scores (Group 1M = 5.12/6), 34% of learners expressed frustration with AI’s lack of cultural adaptability, especially in pronunciation modeling and pragmatic interactions. This limitation was salient in nuanced communication scenarios like accent recognition and politeness strategies in role-play tasks. A recurring concern was accent discrepancies; multiple learners reported the AI favored American pronunciations over British or local English varieties. For instance, one learner (G2-S38, Sichuan) stated, “The AI’s American accent made me feel like a foreigner in my own practice. I started mimicking it unnaturally, and my teacher said I lost my natural flow.” Similarly, LinguaQuest rejected a learner’s indirect request (“Could I trouble you for a menu?”) in a restaurant role-play, favoring direct phrasing (“Give me the menu”), requiring teacher intervention to explain the politeness nuance in Chinese-English interactions. These findings highlight AI-driven language learning’s limitations in addressing sociocultural nuances, reinforcing the importance of human mediation for contextual explanations.

#### 4.2.3 Motivation and affective factors

Motivation was closely tied to learners’ evolving self-concepts and emotional experiences throughout the intervention. Journal entries and interviews suggest Group 1 learners experienced a “novice-to-self-regulated learner” trajectory, while Group 2 showed signs of disengagement due to a “novelty plateau” around Weeks 9–12. While gamified autonomy was initially engaging, it became problematic without guidance. One learner (G2-S19, Week 4) wrote, “I love choosing my own game topics! Picked ‘science’ all month—my vocabulary rocketed.” By Week 12, however, the same learner reported, “Stuck in a loop choosing easy science games. Scared to try ‘politics’—no teacher to help if I fail.” This pattern suggests AI-driven autonomy, while enhancing engagement, must be structured to encourage risk-taking and exposure to diverse content.

Additionally, Group 2 learners frequently described social isolation when practicing with AI for extended periods. One participant (G2-S22, Rural) stated, “At first, talking to AI felt futuristic. By Week 10, it was lonely—like shouting into a void. No laughs, no nods, just ‘Incorrect. Try again.’” In contrast, Group 1’s scaffolded emotional buffering strategies—such as peer debriefs and humor-infused reflections—helped mitigate motivation decay. These findings underscore that sustained engagement requires both cognitive challenge and emotional scaffolding, preventing learners from disengaging due to over-reliance on AI-based repetition.

#### 4.2.4 Challenges and opportunities in AI integration

While 78% of Group 1 learners trusted AI feedback by Week 16, skepticism persisted regarding subjective tasks like essay scoring and pronunciation assessment. A Group 1 learner (G1-S08, Week 14) noted, “I blindly followed AI edits until my teacher said, ‘This sentence lost your original metaphor. Keep your voice!’ Now I debate the AI like a teammate.” Observational data revealed some learners over-relied on AI corrections, leading to stilted phrasing.

Speech recognition challenges also frustrated rural learners, particularly those with dialectal influences. One participant (G2-S29, Week 7) reported, “Shouted ‘She sells seashells’ 10 times—AI heard ‘She cells seashells.’ Gave up and typed it. Felt defeated.” Observational tallies confirmed rural learners required 2.3 times more speech repetitions than urban peers (*p* = 0.013), highlighting accessibility disparities. Beyond these difficulties, some learners expressed anxiety from competitive AI features, with leaderboards encouraging overpractice. One participant (G2-S17, Urban) admitted, “The leaderboard made me play until 2 AM. My eyes burned, but I could not let Team Shanghai fall behind.” In response, Group 1 instructors modified competition structures to emphasize progress over rankings, a strategy absent in Group 2.

These findings suggest that while AI integration offers valuable personalized learning opportunities, it also introduces risks related to trust, overcorrection, and anxiety, necessitating careful instructional design. The qualitative findings contextualize the quantitative trends, reinforcing that sustained learning gains depended on the synergy between AI-driven personalization and human mediation. Learners thrived when teachers reframed algorithmic outputs as dialogic partners rather than authoritative judges, aligning with Self-Determination Theory’s emphasis on autonomy-supportive environments. Conversely, unmediated AI use led to mechanistic practice cycles that eroded motivation, underscoring the importance of structured scaffolding in AI-assisted education.

## 5 Discussion

The present study set out to investigate the long-term effects of AI-driven gamified language learning on Chinese EFL learners’ proficiency, motivation, and engagement, while also exploring the mediating roles of teacher scaffolding and cultural factors. The quantitative findings showed that (1) the AI + Scaffolding group achieved substantially greater IELTS score gains than both the AI Only and Control groups, (2) teacher scaffolding played a pivotal role in nurturing students’ sense of autonomy, competence, and relatedness (Ryan and Deci, 2017), and (3) these improvements were sustained over time, as evidenced by higher retention in delayed post-tests. The qualitative data further illuminated how scaffolding helped learners overcome AI’s limitations—particularly in areas of nuanced language use, cultural adaptability, and motivational support. This section provides a comprehensive discussion of these findings, integrating them with relevant theoretical frameworks and existing research.

### 5.1 Synergy between AI personalization and teacher scaffolding

A core contribution of this study is the elucidation of how AI-driven personalization and teacher scaffolding interact to enhance EFL learning outcomes. Quantitative results indicated that while the AI Only group experienced notable gains, the AI + Scaffolding group outperformed them in both immediate post-test proficiency and longer-term retention. From a theoretical standpoint, this underscores how personalized AI feedback can fulfill learners’ need for competence (Deci and Ryan, 2000; Noels et al., 2019), yet requires human mediation to translate algorithmic outputs into pedagogically meaningful guidance (Chiu et al., 2024; Kessler, 2018). The observed mediation effect of autonomy and competence supports prior research linking self-determination theory (SDT) constructs to robust and sustained language gains (Lamb, 2017).

Qualitative analyses corroborated this synergy, revealing that teachers’ role as “interpreters” of AI feedback addressed key learner frustrations, such as unclear or overly rigid error messages. This dynamic is consistent with Barzilai and Blau’s (2014) argument that scaffolding is vital in digital game-based and AI-assisted contexts to prevent superficial interactions with automated feedback. Indeed, learners reported higher autonomy when teachers helped them refine strategy use or connect AI errors to future classroom goals—aligning with Oxford’s (1990) emphasis on strategy training. Likewise, focusing on the social dimensions of AI-based feedback—through teacher-led reflection and discussion—ensured that learners remained engaged with the technology rather than feeling isolated (García Botero et al., 2021). These findings resonate with Chen et al. (2020) and Li et al. (2025), who emphasize that AI cannot supplant human pedagogy but can instead amplify its effectiveness when integrated thoughtfully.

### 5.2 The role of gamification in sustaining motivation

The gamification component—particularly in the AI-driven groups—appears to have contributed to heightened engagement and motivation, echoing insights from prior game-based language learning research (Dehghanzadeh et al., 2021; Zainuddin et al., 2024). Consistent with Deterding et al.’s (2011) conceptualization of gamification, the presence of points, adaptive challenges, and real-time feedback in LinguaQuest AI triggered “gameful” states (Sailer and Homner, 2020). Learners in both AI groups experienced flow (Csikszentmihalyi, 1990) more frequently than did those in the Control group. However, the significantly higher gains in the AI + Scaffolding group underscore that gamification alone may not suffice to produce sustained growth (Bai et al., 2020); instead, teachers act as catalysts who contextualize game elements to meet curricular needs.

Supporting data from interviews and journals highlighted how teachers in Group 1 enacted “motivational orchestration” by aligning gamified tasks with upcoming course content or by designing supportive competitions (Zou et al., 2023a). This scaffolding moderated the potentially adverse effects of leaderboards—such as heightened anxiety and perfectionism—reported in prior work (Edwards, 2022; Hamari et al., 2014). These findings align with the notion of “pedagogically meaningful gamification,” where technology-based game elements are coupled with deliberate instructional designs (Landers et al., 2018; Hung et al., 2018). By setting clear learning objectives and enabling learners to reflect on progress, teachers created a motivational climate that balanced extrinsic incentives (e.g., leaderboard ranks) with intrinsic satisfaction in achieving personal goals (Deci et al., 1999; Lee, 2016). Ultimately, these results confirm that gamification strategies can be a powerful motivator when they are embedded in a broader pedagogical framework supportive of autonomy, competence, and relatedness (Ryan and Deci, 2017; Dincer and Yesilyurt, 2017).

### 5.3 Cultural and linguistic considerations

A salient qualitative finding was the friction some learners experienced regarding accent recognition and pragmatic variations—particularly among those from rural backgrounds and those whose accents differed significantly from the AI’s U. S.-centric phonetic model. These insights corroborate prior observations that AI language tools often lack socio-cultural adaptability (Chen and Zhao, 2022; Schmidt and Strasser, 2022), an issue that can cause frustration and potentially lower engagement (Rusmiyanto et al., 2023). Studies of advanced speech recognition systems have similarly revealed biases toward standardized accents, underscoring the need for culturally responsive AI design (Aijun, 2024; Huang X. et al., 2023).

In contexts such as China’s multilingual landscape, teacher scaffolding becomes even more critical. In line with Vygotskian theory (Vygotsky, 1978), the instructor mediates cultural mismatches by explaining how pragmatic norms and accent variations are interpreted in both local and global English contexts. Additionally, teachers can incorporate real-life examples of Chinese English usage (Huang J. et al., 2023; Huang X. et al., 2023), ensuring that learners do not over-conform to AI feedback at the expense of more natural or locally relevant language forms (Chamot, 2014). This teacher-facilitated cultural bridging is consistent with calls for more “glocalized” AI solutions that respect local linguistic identities (Hofstede, 2011).

Beyond human mediation, our findings underscore critical directions for the development of AI systems themselves. Future AI tools should be engineered with explicit consideration for linguistic diversity, incorporating a wider range of non-Western English accents in their speech recognition models and offering adaptable pragmatic feedback. This could involve developing localized linguistic corpora derived from diverse global Englishes, allowing for user-customizable accent preferences, or integrating region-specific communicative norms into their algorithms. Such culturally responsive AI tools would empower learners to develop diverse English proficiencies without feeling pressured to conform to a single linguistic standard, thereby enhancing both usability and motivational alignment for non-Western learners.

### 5.4 Teacher mediation and self-regulated learning

Another novel aspect of our findings is the evidence that teacher mediation not only addresses immediate technical or linguistic issues but also fosters learners’ self-regulation and reflective practices over time (Muthmainnah et al., 2024). While AI-based personalization can supply adaptive learning paths (Ayeni et al., 2024; Li et al., 2024), it is ultimately the human teacher who encourages students to (a) interpret feedback more deeply, (b) select challenging tasks rather than “safe” options, and (c) persist in tasks despite algorithmic errors or ambiguous feedback. The result is a shift from “novice to self-regulated learner,” as described by the participants in Group 1—mirroring how teacher support for metacognitive processes leads to more robust and sustainable motivation (Wei, 2023; Yildirim et al., 2023).

These outcomes parallel existing research that underscores the importance of scaffolding for developing autonomy and strategic competence (Chiu et al., 2024; Sung et al., 2017). AI alone can inadvertently encourage repetitious task selection, or what some participants described as “looping through easy topics.” Without teacher intervention, learners may avoid risk-taking (Jeon, 2022), which is critical for language development and consistent with Krashen’s (1982) emphasis on pushing beyond one’s comfort zone for comprehensible input. By contextualizing AI tasks within broader language goals, teachers promote a cycle of reflection, strategy adjustment, and self-assessment, which aligns with self-regulated learning frameworks (Oxford, 1990; Pintrich and De Groot, 1990 as cited in Fryer et al., 2020).

### 5.5 Addressing limitations of AI: trust, overcorrection, and anxiety

Despite the notable benefits of AI for personalization and immediate feedback, several challenges persist that can undermine the learning experience, particularly when teacher scaffolding is absent or minimal. First, some learners expressed skepticism toward AI’s ability to evaluate subjectively complex language tasks (e.g., metaphorical expressions or cultural politeness). This concern reflects a broader issue of “algorithmic trust” in educational contexts, where students may doubt machine-generated feedback on nuanced human communication (Aijun, 2024; Becker, 2023). Our findings echo prior calls for educators to function as trusted intermediaries—validating or contesting AI outputs (Holmes et al., 2023; Sharifuddin and Hashim, 2024).

Second, overcorrection emerged as a theme, where learners uncritically adopted AI’s suggestions at the expense of personal expression. Similar observations have been made in research on automated writing evaluation systems, underscoring the potential for stilted or formulaic language use if learners are not guided to critically evaluate suggestions (Fryer et al., 2020; Warschauer and Grimes, 2008). Indeed, teacher feedback can highlight the creative or stylistic elements that AI feedback often overlooks (Rad et al., 2023).

Third, competitive features such as global leaderboards triggered anxiety and, in some cases, excessive practice that led to burnout—particularly in the AI Only group. This phenomenon underscores the dual-edged nature of gamification: while competition can spur engagement, unchecked rivalry can also escalate stress (Hamari et al., 2014; Edwards, 2022). Teacher-led scaffolding was crucial in modulating competitive climates, switching leaderboards to highlight personal growth rather than peer comparison. By doing so, instructors aligned competition with SDT principles that emphasize autonomy-supportive and competence-affirming feedback (Deci et al., 1999; Reeve and Tseng, 2011).

## 6 Conclusion and implications

This study confirms that integrating AI-driven personalization with teacher scaffolding yields robust, sustained gains in EFL proficiency and learner motivation. Our AI + Scaffolding group consistently outperformed others in IELTS scores and maintained these gains, highlighting the value of human support alongside technology. Interviews and observations further revealed how teachers helped students overcome AI’s shortcomings, especially regarding cultural nuances and practical communication skills. These results underscore the need for a balanced blend of technology and teaching, where instructors guide students to effectively use AI tools.

Based on these findings, teachers are central to optimizing AI-enhanced language learning. Educators should actively serve as “AI Interpreters,” mediating and contextualizing AI feedback. This means translating algorithmic outputs (e.g., “72% accuracy on past tense”) into pedagogically meaningful guidance, explaining AI-flagged errors, or even overriding AI suggestions when cultural or pragmatic nuances are overlooked. To enhance this, teachers can facilitate weekly feedback reviews, guiding students to collectively analyze AI corrections, clarify rationales, and practice applying them to speaking or writing. Furthermore, prioritizing metacognitive scaffolding is crucial. Teachers should encourage learners to reflect on their AI interactions with questions like, “How did AI feedback change your strategy?” or “What did you learn from AI that you would not have learned otherwise?” This fosters active evaluation and application of AI insights. Providing professional development (e.g., workshops, collaborative training) also equips teachers to effectively leverage AI analytics, address common challenges like accent recognition, and scaffold self-regulated learning strategies.

When designing AI-enhanced learning environments, balancing competition with collaboration is essential. While gamified elements like leaderboards motivate, they risk increasing stress or encouraging excessive practice that prioritizes points over genuine skill development. To mitigate this, educators should integrate competitive features with collaborative activities (e.g., peer editing, joint projects) alongside gamified AI features, ensuring motivation without allowing competition to dominate the learning experience. Similarly, teachers must guide student autonomy rather than simply granting it. While AI platforms offer choice, proactive guidance is needed to encourage learners to explore diverse content and challenge themselves. Teachers can achieve this by setting clear learning goals, strategically helping students select optimally challenging tasks, and regularly checking in to adjust goals and offer targeted support.

Finally, championing cultural responsiveness is paramount. Educators should be aware of AI’s linguistic biases (e.g., accent favoritism) and supplement AI practice with real-world examples of diverse English varieties and pragmatic uses. This helps students understand that language is fluid and culturally situated, not just a rigid set of rules dictated by an algorithm. Institutions should prioritize AI tools that accommodate diverse linguistic and cultural expressions, while teachers can adapt tasks to better reflect student real-world language use by adjusting AI prompts to include familiar contexts or daily phrases.

## 7 Limitations and future research

A notable limitation of this study, especially concerning engagement assessment, is its reliance on AI usage logs. While these logs provided valuable quantitative data on interaction frequency and duration, they do not inherently capture the depth or quality of learning. For instance, students might spend extended periods on tasks passively or complete them quickly without fully understanding the content, particularly in gamified settings where extrinsic rewards can drive behavior over intrinsic interest. While our study mitigated this by incorporating rich qualitative data from interviews, observations, and reflective journals for a comprehensive understanding of student engagement, future research could strengthen this by including additional measures like qualitative assessments of student work or direct observations of engagement quality, offering a more nuanced view of AI tool engagement beyond surface-level metrics.

This study also focused on intermediate-level learners at three universities, which may limit how well these findings generalize to other proficiency levels or age groups. While our 16-week intervention and four-week delayed post-test offered insight into short-term retention, a longer follow-up would provide a clearer picture of AI tool use after formal coursework ends. Moreover, relying on a specific AI platform suggests future investigations should explore other technologies and contexts for similar patterns. Further research could also examine teacher scaffolding methods more closely through detailed classroom observations or collaborations with educational technology developers to design adaptive features responding to learners’ emotional cues and cultural backgrounds.

Building on these findings, future research should rigorously explore the nuances of AI-human integration to maximize its potential in diverse educational settings. One critical area involves conducting extended longitudinal studies, potentially over multiple semesters or years, to observe the long-term durability of motivation and proficiency gains. This would assess the transferability of AI-supported skills to real-world communicative contexts beyond controlled learning environments, by following students for months after formal coursework concludes to track continued AI tool use and retention of learned gains. Another crucial direction is to investigate AI-human integration’s effectiveness across diverse linguistic contexts (e.g., other foreign languages or second language acquisition settings) and varied technological setups (e.g., virtual reality, mixed reality, or multimodal AI for complex communication). Furthermore, examining different pedagogical environments, like high schools, vocational training programs, or non-English specific courses, would provide valuable insights into the broader applicability of these findings.

Future studies should also systematically examine effective models for preparing educators to critically evaluate AI tools, interpret analytics, and implement nuanced scaffolding strategies that respond to individual learner needs and cultural backgrounds. This could involve detailed classroom observations and collaborations with educational technology developers to design adaptive features that actively respond to learners’ emotional cues and cultural differences, ultimately aiming for less biased and more useful feedback. Finally, investigating how specific learner characteristics (e.g., learning styles, personality traits, or prior digital literacy levels) interact with different AI features and teacher scaffolding approaches would further inform more tailored learning experiences. Ultimately, this research offers a foundational step toward understanding the “human touch” required to fully realize transformative potential of AI in language education, paving the way for more effective, equitable, and sustainable learning ecosystems.

## Data Availability

The data analyzed in this study is subject to the following licenses/restrictions: the datasets generated and analyzed during this study are not publicly available due to institutional restrictions and participant confidentiality agreements. However, de-identified data may be made available upon reasonable request from the corresponding author, subject to ethical approval. Requests to access these datasets should be directed to Yi Ma, mayi@fosu.edu.cn.
